# Long-Term Changes in Refractive Error and Clinical Evaluation in Partially Accommodative Esotropia after Surgery

**DOI:** 10.1371/journal.pone.0166695

**Published:** 2016-12-09

**Authors:** Shin Yeop Oh, Ju-Yeun Lee, Kyung-Ah Park, Sei Yeul Oh

**Affiliations:** 1 Department of Ophthalmology, Samsung Changwon Hospital, Sungkyunkwan University School of Medicine, Changwon, Korea; 2 Department of Ophthalmology, Samsung Medical Center, Sungkyunkwan University School of Medicine, Seoul, Korea; University of Illinois at Chicago, UNITED STATES

## Abstract

We investigate the changes in refractive error and clinical evaluation in partially accommodative esotropia(PAET) after surgery. A total of 68 patients PAET who received at least 2 years of follow-up after surgery were enrolled in this study. We performed a retrospective study in patients who underwent unilateral or bilateral medial rectus recession for a non-accommodative component of PAET between January 2005 and March 2013. Patients were divided into groups according to the presence of dominancy (dominant, non-dominant, alternative eye), and presence of amblyopia (amblyopic, fellow, normal eye). Changes and changing pattern in SE refractive error were analyzed in all patients and compared between groups. Patients were divided into two groups, those weaned off of hyperopic glasses and those who continued using them, then factors that significantly influenced the continued use of glasses were analyzed. The changes and changing pattern in SE refractive error according to time after operation and presence of amblyopia or dominancy. The mean length of follow-up was 4.89±1.74 years after surgery and the mean change in SE refractive error rate per year was -0.284±0.411 diopters (D). The pattern of changes in the mean SE refractive error for those with dominant, non-dominant, and alternative eyes was not significantly different (p = 0.292). The pattern of changes in the mean SE refractive error for those with amblyopic, fellow, and normal eyes was significantly different (p = 0.0002). Patients were successfully weaned off of hyperopic glasses at an average age of 9.41±2.74 years. The average SE refractive error in the group weaned off of hyperopic glasses was significantly lower than that in the group maintained on hyperopic glasses (p = 0.0002). The change of SE refractive error in amblyopic eyes decreased less than that in fellow or normal eyes, which may be correlated with the presence of amblyopia. Patients with a smaller esodeviated angle without hyperopic correction, a lower degree of hyperopia, and who were older at the time of disease onset were discontinued from hyperopic glasses sooner after surgery.

## Introduction

Accommodative esotropia (AET) usually begins at the age of 2–3 years as the result of uncorrected hyperopia, but may also occur in infancy or later in life. It is divided into fully accommodative esotropia (FAET) and PAET. FAET is corrected with hyperopic glasses only; PAET is treated with glasses for the accommodative component and surgery for the remaining non-accommodative component [1,2]. In normative cohorts, children typically undergo a rapid decrease in hypermetropic refractive error between 3 and 9 months, followed by a long period of near-emmetropia from 1–5 years of age [3,4].

The degree of hyperopia in patients with AET increases until the age of 6–7 years and then rapidly decreases until approximately 14 years old, unlike hyperopia in the general population [5,6]. In addition, the extent of the decrease is relatively small, and the normal emmetropisation process that occurs in nonstrabismic patients does not occur in most patients with accommodative esotropia [7,8].

On the other hand, patients with infantile esotropia (IET) exhibit a different pattern of refractive development than do normative cohorts. After the age of 5 years, patients with IET are similar to their normative counterparts, in that both show myopic shifts after 7 years of age and those with the greatest initial extent of hypermetropia show the greatest degree of myopic shift [9].

The natural history and change of refractive error in FAET and IET has been studied; however, none of the prior reports have examined the change in refractive error in PAET patients after surgical treatment. Although the esodeviation angle decreases after surgery, patients with PAET wear hyperopic glasses continuously until emmetropisation and resolution of PAET occur. It is not yet clear whether patients with PAET will be able to eventually discontinue the use of glasses entirely at some point after surgery.

In this study, we reviewed the long-term changes in refractive error and clinical outcomes in patients with PAET who were treated surgically. In addition, we evaluated the change in refractive error in amblyopic eyes after surgical treatment. Therefore, we provide useful clinical information, especially regarding the change in refractive error in PAET patients after surgery.

## Methods

This study was approved by the ethics committee of the Samsung Medical Center Institutional Review Board. Patient records were anonymized and de-identified prior to analysis.

### Patients

We retrospectively reviewed the medical records of patients with PAET. We performed a computerized search of the records of patients with convergent concomitant strabismus as defined by the *International Classification of Diseases*, *Tenth Revision* who were examined at Samsung Medical Center between January 2005 and March 2013. Patients with hyperopia and a corrected esotropia of more than 10 prism diopters (PD) at both distance and near with the use of full cycloplegic hyperopic correction were considered for inclusion in this study. Patients with decompensated AET or a follow-up interval of less than 2 years were not included in the study. Patients with developmental delays, previous extraocular muscle surgery, any form of neurologic impairment, or other diseases of the visual pathways were also excluded.

### Preoperative measurements

At the initial visit, all patients underwent a full ophthalmologic assessment, including visual acuity testing, cycloplegic refraction, slit-lamp examination, and fundus examination. Ocular alignment status was evaluated and stereoacuity was tested with the Titmus stereo test (Stereo Optical Co., Chicago, IL, USA). Refraction measurements were performed using retinoscopy after administration of 1% cyclopentolate and 0.5% tropicamide. The spherical equivalent (SE) was calculated as the sphere plus half a cylinder. In older children, ocular alignment was tested by prism alternate cover testing at 6 m fixation and 33 cm fixation; the Krimsky or Hirschberg light reflex tests were used in preverbal children. All tests for ocular alignment were performed with and without correction of refractive error. During the follow-up period, cycloplegic refraction tests and evaluation of ocular alignment status were generally performed every 6 months.

### Surgical procedure and postoperative measurements

Patients with PAET underwent unilateral or bilateral medial rectus muscle recession surgery for a non-accommodative component of >15 PD after correction with glasses for the accommodative component by one experienced surgeon (SYO). Spectacles were prescribed on the basis of cycloplegic refraction after a diagnosis of PAET. The entire refractive error, including astigmatism, was fully corrected at the initiation of spectacle treatment. After this, if a change in deviation was not observed for at least 1 year, spectacles undercorrected by -0.25 to -0.50 D based on cycloplegic refraction testing on each visit were prescribed at the discretion of the physician (SYO). During the follow-up period, successful withdrawal from hyperopic glasses, based on a patient achieving complete emmetropisation and maintaining orthotropia, was indicated when a patient maintained orthotropia within ±8 PD without hyperopic glasses for more than 6 months.

### Statistical analyses

The myopic progression of the refractive error was defined as the difference between the final SE refractive error and the preoperative SE, and the mean rate of myopic shift per year was defined as the progression divided by the follow-up period from the preoperative exam to the final visit (D/year).

Longitudinal changes in SE refractive error were evaluated according to age and preoperative hyperopia as well as presence or absence of amblyopia and a dominant eye.

Statistical analyses were performed by an independent statistician using the Statistical Analysis System version 9.4 (SAS Institute Inc, Cary, NC, USA). Analyses associated with longitudinal changes in SE refractive error were performed with a univariate Generalized Estimating Equation (GEE) test. Demographics and differences between the groups were compared using a T-test and Wilcoxon Mann-Whitney U-test. A p-value less than 0.05 was considered statistically significant.

## Results

A total of 68 patients (36 males and 32 females) were included in this study, and the mean length of follow-up was 4.89±1.74 years after surgery. The average age of onset was 1.82±1.43 years (range, 0.5 to 5.5 years) and the average age at the time of the operation was 4.63±2.49 years (range, 1.5 to 11.3 years). The preoperative mean SE refractive error was 3.38±1.92 D and the angles of deviation without correction were 48.82±14.66 PD (distance) and 50.44±15.13 PD (near). 21 of the 68 patients (30.9%) had amblyopia and 12 (17.6%) had alternative fixation on the initial follow-up. The average age at which 25 of the 68 (36.8%) patients were successfully weaned from hyperopic glasses was 9.41±2.74 years (range, 4.3 to 16.3 years) and the postoperative latency until patients were weaned from hyperopic glasses was 3.49±2.16 years (Table 1).

**Table 1:** Demographics of patients with partially accommodative esotropia given surgery.

Parameters	
Total patients (n)	68
Male: Female (n)	36: 32
Age of onset (years)	1.82 ± 1.43
Age at surgery (years)	4.63 ± 2.49
Duration of follow-up (years)	4.89 ± 1.74
Preoperative SE refractive error (D)	3.38 ± 1.92
Preoperative angle of deviation at distance and near (without correction) (PD)	48.82 ± 14.66 / 50.44 ± 15.13
Amblyopic: Fellow: Normal eyes (n)	21: 21: 94
Dominant: Alternative fixation eyes (n)	56(od: 30, os: 26): 12
Weaning: Maintaining of hyperopic glasses (n)	25: 43
Age of weaning of hyperopic glasses (years)	9.41 ± 2.74
Postoperative duration until weaning of hyperopic glasses (years)	3.49 ± 2.16

Values are presented as mean ± SD.

SD = standard deviation; SE = spherical equivalent; PD = prism diopter; D = diopter.

The SE refractive error decreased gradually with age (p<0.001) and the mean change in the error per year was -0.28±0.41 D. The preoperative mean SE refractive error was 3.38±1.92 D; it was 3.15±1.97 D at 6 months postoperatively, 2.87±1.91D after 1 year, and 2.55±1.90 D after 2 years in all 68 patients. The postoperative mean SE refractive error was 2.24±1.89 D after 3 years in 67 patients, 2.05±1.96 D after 4 years in 46 patients, and 1.96±2.09 D after 5 years in 23 patients (Table 2).

**Table 2:** The change in mean SE refractive error after surgery.

Follow-up period						
	Pre-op	1year	2years	3years	4years	5years
Mean SE refractive error(D)	3.38±1.92	2.87±1.91	2.55±1.90	2.24±1.89	2.05±1.96	1.96±2.09
No. of patients (n)	68	68	68	67	46	23
Postoperative change of SE refractive error rate per year: -0.28±0.41 D						

Values are presented as mean ± SD.

SD = standard deviation; Pre-op = pre-operation; D = diopter; No = number.

**Fig 1:**
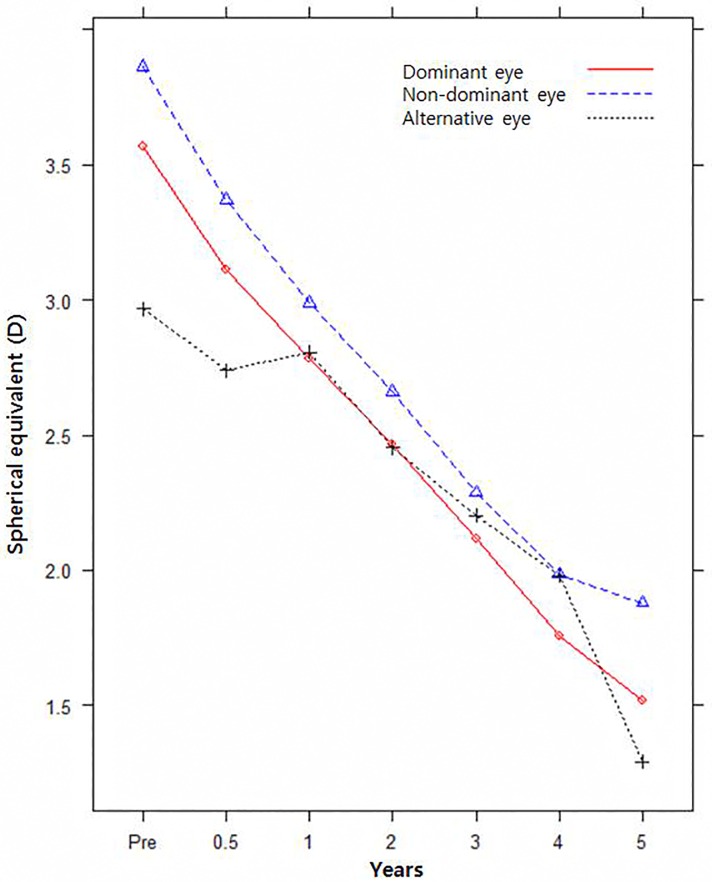
Pattern of change of mean SE refractive error for dominant/non-dominant/alternative fixation eyes.

**Fig 2:**
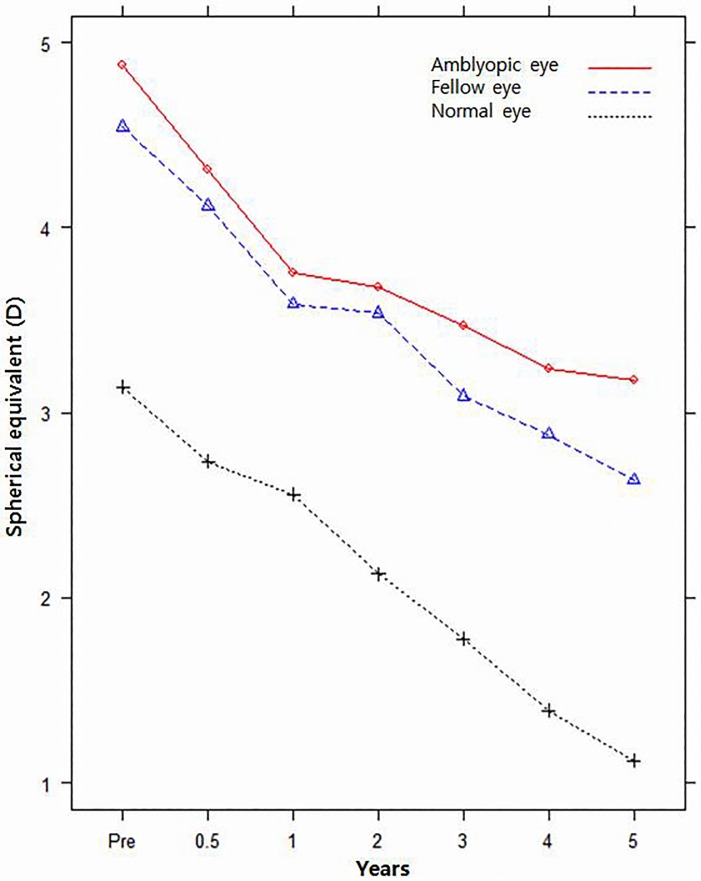
Pattern of change of mean SE refractive error for amblyopic/fellow/normal eyes.

The postoperative mean SE refractive error was significantly different (p = 0.026), but the pattern of change was not significantly different (p = 0.292) for dominant/non-dominant/alternative eyes (Fig 1). The mean SE refractive error in non-dominant eyes was more hyperopic than that in alternative eyes, but was not different from that in dominant eyes (p = 0.0456, p = 0.1815). The mean SE refractive error of dominant eyes was more myopic than that of non-dominant eyes (p = 0.0059) (Table 3). The postoperative mean SE refractive error and pattern of change were significantly different between amblyopic, fellow, and normal eyes (p = 0.0001, p = 0.0002) (Fig 2). Amblyopic eyes and fellow eyes were more hyperopic than were normal eyes (p = 0.0002, p = 0.0062). The mean SE refractive error of amblyopic eyes was more hyperopic than that of fellow eyes, but the statistical significance of this finding is marginal (p = 0.0489) (Table 4).

**Table 3:** The difference in postoperative SE refractive error in dominant/non-dominant/alternative eyes.

	Dominant eye	Non-dominant eye
Alternative eye	0.60 ± 0.45	0.90 ± 0.45
p-value	0.1815	0.0456
Non-dominant eye	-0.30 ± 0.11	
p-value	0.0059	

Values are presented as mean ± SD.

SD = standard deviation; PD = prism diopter; D = diopter.

P values were obtained using the univariate Generalized Estimating Equation (GEE) test.

**Table 4:** Difference in postoperative SE refractive error in amblyopic/fellow/normal eyes.

	Amblyopic eye	Fellow eye
Normal eye	1.74 ± 0.46	1.40 ± 0.51
p-value	0.0002	0.0062
Fellow eye	0.39 ± 0.20	
p-value	0.0489	

Values are presented as mean ± SD.

SD = standard deviation; PD = prism diopter; D = diopter.

P values were obtained using the univariate Generalized Estimating Equation (GEE) test

Meanwhile, the pattern of change of SE refractive error was not significantly different between dominant/non-dominant eyes and amblyopic/fellow eyes (p = 0.6658 and p = 0.0646, respectively).

25 of the 68 patients (36.8%) discontinued wearing hyperopic glasses for esotropia at a mean age of 9.41±2.74 years. In comparison with the groups weaning off of and maintaining the use of hyperopic glasses, the preoperative SE refractive error was 2.9±1.6 D / 4.5±2.6 D (p = 0.0002), age of onset was 2.49±1.65 years / 1.4±1.07 years (p = 0.005), age at operation was 5.93±2.01 years / 4.35±2.58 years (p = 0.001), and angle of deviation at distance was 43.60±14.61 D / 51.86±13.97 D (p = 0.011) and at near was 44.80±14.84 D / 53.72±14.44 D (p = 0.010), which are all significantly different (Table 5). The results of stereopsis tests (Titmus tests) taken during the follow-up period were available for 50 patients (73.5%). 10 patients (20%) had stereopsis of 100 seconds of arc or better, 28 patients (56%) had 100 to 400 seconds of arc, 9 patients (18%) had 800 or more seconds of arc, and the remaining 3 patients (6%) had no stereopsis.

**Table 5:** Comparison between those weaned from and those maintained on hyperopic glasses.

Parameters	Weaning (25)	Maintaining (43)	p-value
Age of onset (years)	2.49 ± 1.65	1.4 ± 1.07	0.005
Age at surgery (years)	5.93 ± 2.01	4.35 ± 2.58	0.001
Preoperative angle of deviation at distance (without correction) (PD)	43.60 ± 14.61	51.86 ± 13.97	0.011
Preoperative angle of deviation at near (without correction) (PD)	44.80 ± 14.89	53.72 ± 14.44	0.010
Preoperative mean SE refractive error (D)	2.9 ± 1.6	4.5 ± 2.6	0.0002

Values are presented as mean ± SD.

SD = standard deviation; PD = prism diopter; D = diopter.

P values were obtained using the Wilcoxon Mann-Whitney test

## Discussion

AET is a convergent deviation that is the result of abnormal activation of the accommodation reflex required to overcome the blurred vision associated with uncorrected hypermetropia [10]. Treatment typically consists of hypermetropic spectacle correction followed sometimes thereafter with surgery for those with a significant non-accommodative element, as in PAET. The change in refractive error in AET has been several studied before; however, long-term changes have not. Thus, we describe the change in SE refractive error in PAET after surgery.

A value of -0.16±2.0 D/year was reported by Berk, whereas Repka found a decline in hyperopia of -0.12 D/year in children over 7 years of age with AET [11,12]. In our study with 68 patients, a myopic shift of -0.28±0.41 D was found; this value is a little higher than those reported by others. We suggest that myopic shifts may be the result of complex interactions between genetic and environmental factors. Also animal studies have demonstrated alterations in optical blur, resulting in a myopic shift during ocular development [13,14]. Some investigators have argued that full correction of hyperopia may inhibit emmetropisation, thereby prolonging time in glasses and delaying the resolution of esotropia [15,16]. We hypothesize that retinal blur and an increased amplitude of divergence caused by the undercorrection of hyperopia may play a part in the pathophysiology of myopia. The differences may reflect the fact that we did not prescribe full hyperopic correction glasses, but the weakest hyperopic correction that would allow for the best corrected visual acuity with fusion. Another possible explanation is that there are racial differences between the study populations. Although this study not compare the differences of refractive error on surgical and non-surgical group, additionally, we should considerate effect of strabismus surgery on the refractive and astigmatic status. Previous study [17] described that horizontal rectus muscle surgery tended to induce a transient, statistically significant change in the SE refractive error, with a myopic shift that was clinically not significant (at 4–6 weeks postoperative changes -0.23±0.78 D, at 4–6 months postoperative changes -0.14±0.85 D). Also, other study [18] reported that significant myopic shift and astigmatic change were determined in the SE refractive error in the early postoperative period (first month); however, these changes disappeared in the long term (at the postoperative first year). Therefore, in our opinion, short-term SE refractive error changes do not affect directly change or weaning of hyperopic glasses.

The SE refractive error of alternative eyes was slightly more hyperopic than that of non-dominant eyes during the follow-up period, but more hyperopic than that of dominant eyes from 2 to 4.5 years postoperatively. Meanwhile, the postoperative mean SE refractive error was significantly different but the pattern of change was not significantly different between dominant, non-dominant, and alternative eyes. We suggest that dominancy is correlated with the degree of hyperopia but has less to do with patterns of change in SE refractive error. The postoperative mean SE refractive error and pattern of change were significantly different between amblyopic, fellow, and normal eyes. The mean SE refractive error of amblyopic eyes was more hyperopic than that of non-amblyopic fellow and normal eyes during the complete 5 year follow-up period. In agreement with the results of previous studies, a smaller decrease in hyperopia was noted in amblyopic eyes compared with non-amblyopic fellow and normal eyes [19–21]. There is some evidence that amblyopia may affect emmetropisation in strabismic amblyopia and moderate to high hypermetropia [20]. In amblyopic eyes, the initial mean SE refractive error was more hyperopic and the pattern of change of myopia was less than in fellow or normal eyes. Thus, we suggest that the presence of amblyopia is correlated with the degree of hyperopia and the pattern of change of SE refractive error in PAET. In contrast, the pattern of change of the mean SE refractive error was not significantly different regardless of dominancy because dominancy was common in patients without amblyopia.

The SE refractive error was significantly different in the dominant vs. non-dominant eye and the amblyopic vs. fellow eye in one patient. However, the pattern of change of SE refractive error in the dominant vs. non-dominant eye and the amblyopic vs. fellow eye was not significantly different. This result means that refractive error in that patient changed similarly regardless of dominancy or amblyopia, even though the extent of refractive error was different in both eyes.

Black found that 13% of patients (37/285) with AET discontinued the use of glasses for esotropia at a mean age of 11.6 years (range, 7–17 years) during a mean follow-up period of only 1.9 years [5]. Rutstein and Marsh-Tootle reported similar findings in 5 of 39 AET patients (12.8%) who were orthotrophic and did not wear glasses in an average follow-up period of 9.5 years [7]. The current study reports a relatively higher percentage of patients who were able to discontinue glasses for distance esotropia; 25 of 68 (36.8%) discontinued the use of hyperopic glasses for esotropia at a mean age of 9.41±2.74 years. Those weaned from hyperopic glasses also did so at an earlier age and more patients than other previous studies in AET. This result is possibly explained by the long duration of follow-up and the moderate degree of hyperopia in PAET as contrasted with AET. And Factors associated with the discontinuation of hyperopic glasses for esotropia in this study include lower initial SE refractive error, lower initial angle of deviation at distance/near, and higher age of onset. Black also noted that children with a lower initial degree of hypermetropia were more likely to experience a resolution of esotropia, and Brian reported that being weaned from spectacles for esotropia was associated with lower initial hyperopic error [5,22]. Thus, we show that earlier weaning from hyperopic glasses is associated with lower SE refractive error, lower initial angle of deviation, and higher age of onset.

Stereopsis was present in 50 patients, 47 of whom (94%) achieved stereopsis (3000 s of arc or better) at the final follow-up and 20 of whom (40%) had a stereopsis of 200 s of arc or better. Another study reported that most PAET patients (94.6%) and AET patients (98.3%) showed an equivalent improvement in stereopsis at cessation of hyperopia. This positive result was brought about by only including patients successfully discontinued from corrective glasses and those wearing the weakest possible hyperopic glasses that would achieve the best vision while maintaining fusion [23]. When compared with previous studies on AET and IET, stereopsis in our study was better in those with IET and worse in those with AET. This result is explainable by the fact that other studies included patients with alignment during the first 2 years of life, which is associated with a higher prevalence of stereopsis [24,25]. Many authors have determined that patients with AET may end up with a near-normal level of stereopsis if they achieve orthotropia through the correction of hyperopia, but some children with AET continue to have abnormal stereopsis despite successful optical realignment [26,27]. In other words, better stereopsis results are present in patients with AET because AET usually occurs at the age of 2–3 years, unlike IET, and orthotropia can be treated with refractive correction. We surmise that patients in our study showed less of an improvement in stereopsis than do those with AET because hyperopic correction was not enough to maintain orthotropia without surgery and then period of misalignment are remained before surgery.

There are some limitations to the present study. First, this is a retrospective study and it is difficult to control for a selection bias. However, patients were observed for at least 2 years, with an average of 4.89 years, which is quite a long follow-up period. Second, a relatively small number of patients met the inclusion criteria and all of the subjects were East Asian; thus, the results may not be generalizable to other ethnic groups. Third, occlusion treatment was not related to SE refractive error in amblyopic patients. And we could not considerate that surgery of horizontal muscle has significant short-term effects on astigmatism or refractive error in patients of PAET. The larger study with surgical effects needed to extend our results, overcome the limitations of the study and carry out improvements suggested by other studies.

In conclusion, the present study represents the first long-term follow-up results for patients with PAET who received surgical treatment and correction with hyperopic glasses. This study revealed that the SE refractive error decreases after surgery and the myopic shift in SE refractive error in amblyopic eyes is lower than that of non-amblyopic fellow eyes or normal eyes in patients with PAET. Also, the initial mean SE refractive error was more hyperopic in amblyopic eyes, and we suggest that the presence of amblyopia is correlated with the pattern of change of SE refractive error in PAET. In addition, we showed that earlier weaning from hyperopic glasses is associated with lower SE refractive error, lower initial angle of deviation, and higher age of onset, and patients with PAET achieved good stereopsis results after surgery. The effect of amblyopia on changes in SE refractive error needs further evaluation. We believe that our results can help in the management of PAET and will be an important guide for those treating PAET patients with hyperopic glasses after surgery.

## Supporting Information

S1 File This data used for analysis in this study. (XLSX)
